# Tetramer guided, cell sorter assisted production of clinical grade autologous NY-ESO-1 specific CD8^+^ T cells

**DOI:** 10.1186/s40425-014-0036-y

**Published:** 2014-10-14

**Authors:** Seth M Pollack, Robin L Jones, Erik A Farrar, Ivy P Lai, Sylvia M Lee, Jianhong Cao, Venu G Pillarisetty, Benjamin L Hoch, Ashley Gullett, Marie Bleakley, Ernest U Conrad, Janet F Eary, Kendall C Shibuya, Edus H Warren, Jason N Carstens, Shelly Heimfeld, Stanley R Riddell, Cassian Yee

**Affiliations:** Clinical Research Division, D3-100 Fred Hutchinson Cancer Research Center, 1100 Fairview Ave, Seattle, WA 98109 USA; Department of Medicine, University of Washington, Seattle, WA USA; Department of Surgery, University of Washington, Seattle, WA USA; Department of Pathology, University of Washington, Seattle, WA USA; Department of Pediatrics, University of Washington, Seattle, WA USA; Department of Orthopedics, University of Washington, Seattle, WA USA; Department of Radiology, University of Alabama, Birmingham, AL USA; Institute for Advanced Study, Technical University of Munich, Munich, Germany; Department of Melanoma Medical Oncology, UT MD Anderson Cancer Center, 7455 Fannin St, Unit 904, Houston, TX 77054 USA

**Keywords:** Adoptive T cell therapy, NY-ESO-1, Synovial sarcoma, Myxoid, Liposarcoma, Immunotherapy, Antigen specific T cells, Tetramer, Influx cell sorting

## Abstract

**Background:**

Adoptive T cell therapy represents an attractive modality for the treatment of patients with cancer. Peripheral blood mononuclear cells have been used as a source of antigen specific T cells but the very low frequency of T cells recognizing commonly expressed antigens such as NY-ESO-1 limit the applicability of this approach to other solid tumors. To overcome this, we tested a strategy combining IL-21 modulation during *in vitro* stimulation with first-in-class use of tetramer-guided cell sorting to generate NY-ESO-1 specific cytotoxic T lymphocytes (CTL).

**Methods:**

CTL generation was evaluated in 6 patients with NY-ESO-1 positive sarcomas, under clinical manufacturing conditions and characterized for phenotypic and functional properties.

**Results:**

Following *in vitro* stimulation, T cells stained with NY-ESO-1 tetramer were enriched from frequencies as low as 0.4% to >90% after single pass through a clinical grade sorter. NY-ESO-1 specific T cells were generated from all 6 patients. The final products expanded on average 1200-fold to a total of 36 billion cells, were oligoclonal and contained 67-97% CD8^+^, tetramer^+^ T cells with a memory phenotype that recognized endogenous NY-ESO-1.

**Conclusion:**

This study represents the first series using tetramer-guided cell sorting to generate T cells for adoptive therapy. This approach, when used to target more broadly expressed tumor antigens such as WT-1 and additional Cancer-Testis antigens will enhance the scope and feasibility of adoptive T cell therapy.

**Electronic supplementary material:**

The online version of this article (doi:10.1186/s40425-014-0036-y) contains supplementary material, which is available to authorized users.

## Background

The use of antigen-specific T cells for adoptive immunotherapy of patients with advanced cancers is emerging as a promising treatment modality [1-3]. Recent trials using genetic modification of T cells to introduce chimeric antigen receptors (CAR’s) or T cell receptors (TCR’s) and redirect their target specificity to tumor cells can be remarkably effective in reducing tumor burden and providing durable remissions in B cell malignancies and some solid tumors [4-10]. However, the regulatory and logistical hurdles of gene transfer can be prohibitive and issues such as receptor mispairing (for TCRs), target antigen affinity, and serious, occasionally life-threatening, even lethal on-target toxicities remain to be fully addressed [11,12]. Although once fully characterized, these genetically modified approaches may be quite expedient, they first require a priori possession of the relevant receptor sequence from the cognate antibody for a CAR or a T cell clone for a TCR as well as rigorous validation, all of which limit the flexibility of this approach and incur significant obstacles to bringing a cell product to the clinic.

An alternative approach, involves the isolation of circulating endogenous antigen-specific T cells from the peripheral blood (Endogenous T Cell Therapy or ETC) and ex vivo expansion for adoptive transfer. Although these tumor-reactive T cells can be present at very low frequency, the feasibility of isolating and expanding endogenous antigen-specific CTL from the peripheral blood of patients for adoptive therapy has been demonstrated in trials targeting melanocyte antigens such as gp100 and MART-1 in melanoma, with intriguing evidence of clinical efficacy [2,13]. The endogenous frequency of CTL targeting melanocyte antigens such as MART-1 is particularly high (up to 1%) enabling facile generation of MART-1-specific T cells using conventional approaches [2,13,14]. However, for more commonly expressed tumor antigens such as WT-1, MAGE family antigens and NY-ESO-1, the generation of antigen-specific cytotoxic T lymphocytes (CTL) has been labor and resource-intensive and largely unsuccessful on a routine basis due to their relatively rare endogenous precursor frequencies (<1:10,000).

With the exception of MART-1 and some tumor-associated viral antigens, generation of antigen-specific T cells using a conventional approach involving repeated cycles of autologous PBMC stimulation using peptide-pulsed dendritic cells (DC’s) often fails to enrich low frequency tumor-reactive CTL to numbers sufficient for expansion and adoptive transfer.

To address this issue, we previously discovered that the addition of IL-21 during the initial priming period could increase the total number of antigen-specific CD8 T cells by >20-fold and, at the clonal level, enrich for a population of high-affinity CD8 T cells with sustained elevation of CD28 levels and a helper-independent phenotype. This enrichment can be further enhanced by depletion of CD25+ Treg cells from the responder PBMC prior to in vitro stimulation [15-17], leading to a synergistic increase in the yield of antigen-specific CTL to 2-300 fold greater than those cultures that were CD25 replete and not exposed to IL-21 [18,19].

Therefore, to broaden the scope of patients eligible for adoptive therapy and the tumors that can be treated, we chose to target a prototypic cancer-testis antigen, NY-ESO-1, expressed by several solid tumor malignancies, including breast cancer, lung cancer, melanoma, sarcoma and ovarian cancer. We developed a strategy where NY-ESO-1 specific T cells were first enriched through *in vitro* stimulation of CD25 depleted PBMC [17] with peptide pulsed dendritic cells in the presence of IL-21, followed by tetramer guided cell sorting to isolate and expand autologous NY-ESO-1-specific CTL from the peripheral blood of patients with sarcoma under clinically compliant manufacturing conditions.

To determine whether highly avid, oligoclonal NY-ESO-1 specific CD8^+^ T cells recognizing NY-ESO-1 positive tumor cell lines could be consistently isolated from patients who might benefit from NY-ESO-1 targeted therapy, we focused on patients with synovial sarcoma (SS) and myxoid/round cell liposarcoma (MRCL) because these tumors homogenously express NY-ESO-1, often with high intensity [20,21]. We successfully isolated NY-ESO-1 specific T cells from 6 of 6, NY-ESO-1 expressing sarcoma patients using a clinical grade INFLUX cell sorter (Becton Dickson) and propagated these highly enriched populations to sufficient numbers for adoptive immunotherapy.

## Results

### Patient characteristics and leukapheresis yield

Isolation and expansion of NY-ESO-1 specific T cells from leukapheresis products was attempted in six patients with SS (n = 5) and MRCL (n = 1) that expressed NY-ESO-1 in their diagnostic tumor biopsies (Table 1). The median age of these patients was 44 (26-48), which is older than the reported median age for SS patients [22]. Prior to leukapheresis, two of the six patients had received chemotherapy including doxorubicin and ifosfamide (A/I). The remaining four patients underwent leukapheresis before receiving chemotherapy. A range of 5 × 10^9^ – 13.6 × 10^9^ mononuclear cells was obtained by leukapheresis from each of the six patients. The yield did not correlate with prior chemotherapy, suggesting that prior chemotherapy was not a significant barrier to obtaining an adequate leukapheresis collection (Table 1). We depleted CD25^+^ cells from an aliquot of 2 × 10^9^ cells to remove regulatory T cells prior to establishing T cell cultures resulting in a 1-2 log reduction in CD25^+^ cells (data not shown). The average yield after CD25 depletion was 1.34 × 10^9^ cells (range 0.99 to 1.56 × 10^9^).

**Table 1:** **Leukapheresis yield in advanced sarcoma patients**

	**Sarcoma subtype**	**Age**	**Chemotherapy prior to leukapharesis**	**Sites of disease**	**Leukapharesis yield (×10^9)**	**Yield of CD25 depletion (×10^9)**
Patient 1	SS	47	None	Soft tissue, lung, brain	7.11	0.99
Patient 2	MRCL	35	Rtx	Bone, soft tissue, lung	6.9	1.44
Patient 3	SS	48	A/I, HD Ifos, Rtx	Brain, lung	5.14	1.2
Patient 4	SS	46	A/I/Vincristine, Rtx/Ifos	Lung	14	1.365
Patient 5	SS	26	None	Lung, kidney, soft tissue	11.2	1.56
Patient 6	SS	42	None	Recurrent, locally advanced axillary disease	13.76	1.46

Rtx - Radiation therapy; A/I - Adriamycin and Ifosfamide; Gem/tax - gemcitabine and docetaxel; Doxil - liposomal doxorubicin; A/I/DTIC - Adriamycin, Ifosfamide and Decarbazine.

### Production of clinical grade NY-ESO-1 specific T cells by cell sorting

**Figure 1:**
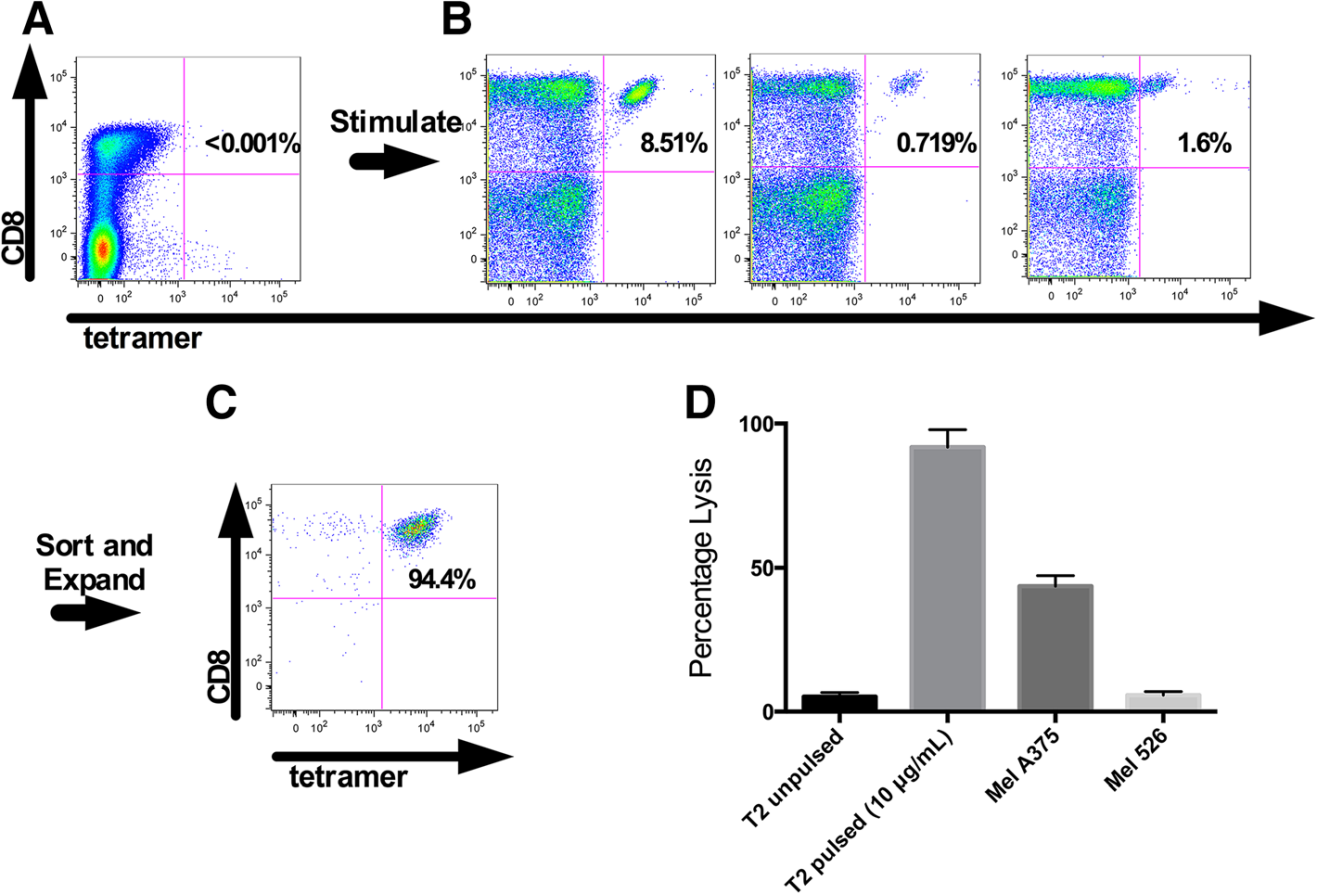
**Representative production of clinical grade NY-ESO-1 specific T cell products from patient 1. A**. No detectable cells are observed with CD8 and Tetramer staining of untreated PBMC from patient 1. **B**. Small CD8^+^ and Tetramer^+^ were observed in 3 wells of three 48 well plates after 2 stimulations using peptide pulsed dendritic cells. **C**. The 3 positive wells were sorted using the clinical grade cell sorted and underwent 2 expansions. CD8 and tetramer staining of the final product is shown. **D**. The final product was able to lyse peptide pulsed targets as well as an endogenously NY-ESO-1 expressing tumor line (NY-ESO-1). Mel 526 is an HLA-A2^+^, NY-ESO-1^−^ tumor line used as a control.

The strategy to isolate NY-ESO-1 specific T cells from PBMC is illustrated in Figure 1. For patient #1, three 48 well plates were plated with T cells and peptide pulsed DC’s. After two stimulations, each individual well was stained with the NY-ESO-1 tetramer and analyzed by flow cytometry. NY-ESO-1 tetramer positive CD8^+^ T cells were not observed in the starting leukapheresis product (detection threshhold <0.01%, Figure 1A), however 3 of the 144 stimulated wells contained NY-ESO-1 tetramer positive T cells at a frequency of >0.5% (Figure 1B). These three wells were pooled and the tetramer binding T cells were sorted, expanded initially in a T25 flask and subsequently in Lifecell Bags in the CPF. The final product was >94% CD8^+^ and NY-ESO-1 tetramer positive and specifically lysed T2 cells pulsed with NY-ESO-1_157-165_ peptide and the NY-ESO-1 expressing melanoma cell line, MelA375 (see Figure 1C and D).

Similar to patient #1, none of the remaining 5 patients had detectable NY-ESO-1 specific T cells by tetramer staining of starting PBMC (data not shown). One or two 48-well plates were seeded with T cells and stimulated with NY-ESO-1_157-165_ peptide pulsed DC’s. After two stimulation cycles, at least three wells (range 3 to 7) from each patient contained NY-ESO-1 tetramer positive cells at a frequency of 0.01-8.05% (Table 2). Overall, the average number of wells per patient that contained NY-ESO-1 tetramer positive cells was 3.8 (out of total of 48-144 wells) and the mean frequency of NY-ESO-1 tetramer positive cells in these wells was 1.67%. This contrasts with the robust response seen with MART-1 where under similar culture conditions typically every well will have >1% tetramer positive cells, and occasional wells will exhibit a robust MART-1-specific response with >20% tetramer positive cells (Additional file 1: Figure S1).

**Table 2:** **Generation of clinical grade products**

	**Number of plates**	**Number positive wells**	**Least % positive sorted**	**Largest % positive (all sorted)**	**Mean % positive**	**Number of wells used for expansion to final product**	**Total cells after mini-REP (×10^6)**	**Bag REP vs. GREX REP**	**Cells started for large volume expansion (×10^6)**	**Final cell number (×10^9)**
Patient 1	3	3	0.62%	8.05%	3.39%	3	230	Bag	33	57.9
Patient 2	1	5	0.57%	1.90%	1.00%	5	73	GREX	68	44.2
Patient 3	1	7	0.01%	5.23%	1.23%	7	224	GREX	25	26.4
Patient 4	2	6	0.11%	3.68%	1.03%	4	157	GREX	19	20.7
Patient 5	2	3	0.07%	1.55%	0.57%	3	331	GREX	32	
Patient 6	2	5	0.11%	7.24%	2.80%	1	169	Bag	18	24.6
Mean	1.8	4.8	0.2%	4.6%	1.7%	3.8	197.3		32.4	34.8

The flow sorted NY-ESO-1-specific T cells were then stimulated with anti CD3 mAb in T25 flasks, which yielded an average of 197 × 10^6^ (range 73 to 331 × 10^6^) T cells after 14 days. Larger volume expansions were then performed in either GREX containers or cell culture bags in the CPF. For two of the patients (#4 and #6), we performed the large volume expansion on T cells sorted from only a fraction of the positive wells. The average bag or GREX REP produced a 1204-fold expansion (range 650-1754 fold). All cell products were expanded to over 2 × 10^10^ cells, and except for patient #5, the final cell product was >85% CD8^+^ and NY-ESO-1 tet^+^ (range 67.2-97.1%) (Table 2 and Additional file 1: Figure S2). Based on these percentages, the absolute number of antigen specific CD8^+^, tet^+^ cells in each product ranged from 16.7 × 10^9^ to 54.7 × 10^9^. Cell doses in prior studies performed by our program that have led to clinical responses used 10^10^ cells/m^2^, suggesting this strategy for isolating and expanding NY-ESO-1 specific T cells can yield therapeutically relevant cell doses.

### Phenotype and function of NY-ESO-1-specific CD8^+^ T cells

Exposure to IL-21 during *in vitro* priming has previously been shown to enrich for a population of CD8^+^ T cells with high affinity recognition of tumor antigen, effector function, and expression of co-stimulatory molecules such as CD28 [18,19]. Phenotype analysis of the final expanded NY-ESO-1 specific T cell products demonstrated expression of CD45RO, CD27 and CD28 on the majority of CD8^+^ T cells, and the absence of CCR7 or CD62L, consistent with an effector memory like phenotype. In almost all cases, a subpopulation of CD127^hi^ also appears in the final T cell product also suggesting a memory-like phenotype (see Additional file 1: Figure S3).

**Figure 2:**
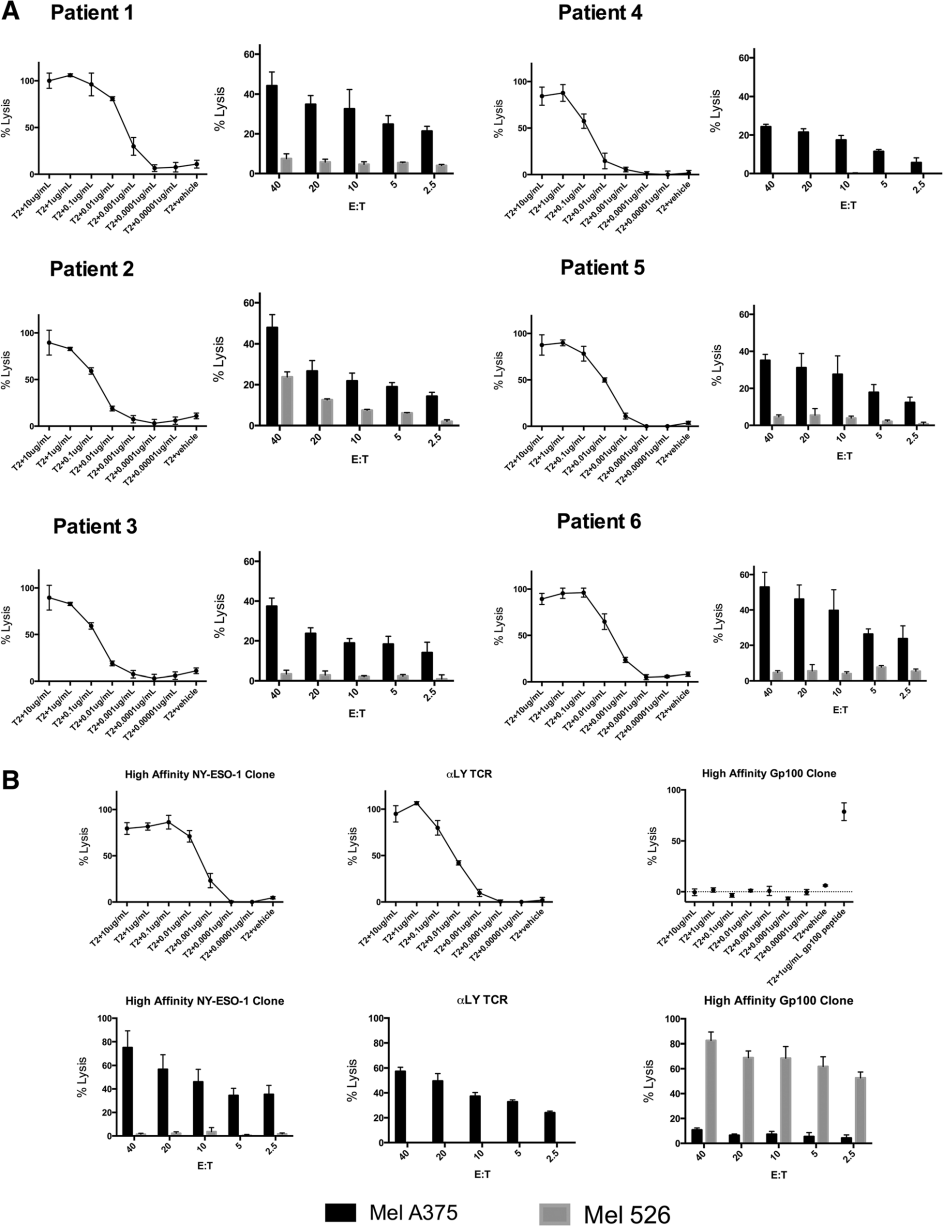
**Functional avidity of NY-ESO-1 specific T cells. A**. NY-ESO-1 specific T cell products from patients #1-#6. The left panel for each patient shows the lysis of T2 cells pulsed with various concentrations of NY-ESO-1 peptide by NY-ESO-1-specific T cells at an effector to target (E:T) ratio of 20:1. The right panel shows lysis of the NY-ESO-1 expressing tumor cell line MelA375 at various E:T ratios. The tumor cell line Mel526 (NY-ESO-1 negative, gp100 positive) is a negative control. **B**. Lysis of NY-ESO-1 peptide pulsed T2 cells and tumor cells by a high affinity NY-ESO-1 T cell clone isolated by our lab, T cells transfected with the αLY TCR and sorted to >80% purity with NY-ESO-1 tetramer, and a gp100_154-162_ specific T cell clone.

We evaluated the function of the NY-ESO-1-specific T cell products by assaying specific lysis of T2 (HLA-A2^+^) targets pulsed with titrated concentrations of NY-ESO-1 peptide as well as the NY-ESO-1^+^ tumor cell line Mel A375. All cell products exhibited specific lysis of T2 cells pulsed with <0.01 μg/ml of NY-ESO-1 peptide and of the Mel A375 tumor cells that endogenously expressed NY-ESO-1 (Figure 2A). The lytic ability of NY-ESO-1 specific CTL generated from the sarcoma patients in this study was comparable to a high affinity NY-ESO-1-specific T cell clones that we previously isolated [23], and to T cells transduced with the high affinity mutant αLY NY-ESO-1 specific TCR and sorted to >80% purity (Figure 2A and B). In response to T2 cells pulsed with NY-ESO-1 peptide, the T cell products from all patients secreted IFN-γ (mean 305 pg/mL, range 143 to 425 pg/mL) and TNF alpha (mean 674.9 pg/mL, range 313.4 to 1113.9 pg/mL) (Additional file 1: Figure S4). In each case, the NY-ESO-1 specific CTL lines were also confirmed to lyse a SS tumor line (SYO-1) and a MRCL tumor line (402) which had been transfected with the gene for A*0201 (Additional file 1: Figure S5).

Thus, the avidity and effector function of antigen-specific CTL derived from the peripheral blood is sufficient to recognize endogenous tumor associated antigens and compares favorably to a clinically relevant high affinity mutant TCR. In order to confirm that NY-ESO-1 specific cells could be generated in patients without NY-ESO-1 expressing tumors, we also made cells through this process in two healthy donors which were also able to recognize peptide pulsed T2’s and endogenously expressed peptide.

### Clonality of products

We next assessed the clonality of the T cell products using TCR Vβ spectratyping. All products were oligoclonal with a range of high intensity peaks from 2 (patient #2) to 15 (patient #4) (Additional file 1: Figure S6). In order to better quantify the TCR composition of these products, deep sequencing of the TCR was performed on patient #1 and patient #4 (Adaptive Biotechnologies). Despite the fact that the cell product from patient #1 was comprised of tetramer positive T cells sorted and expanded from 3 individual 48 wells, the final cell product was almost entirely comprised of a single dominant clone constituting 86.3% of the TCR sequence reads. The next 5 most common sequences comprised 7.3% of the total sequences observed. A total of 235 were observed in the infusion product (Additional file 1: Figure S7). The product from patient #4 combined 4 wells and also contained a dominant clone (85.4% of the sequences). The next most common 6 clones comprised 9.05% of the product and a total of 435 sequences were observed. In composite, our data demonstrates the feasibility and reproducibility of generating a single clone dominant but still oligoclonal, tumor-reactive, NY-ESO-1 specific CD8^+ -^T cells from sarcoma patients using clinical cell sorting technology.

## Discussion

In light of the significant clinical responses observed among patients receiving antigen-specific T cells recognizing melanoma and leukemia-associated antigens, a means to target more commonly expressed tumor-associated antigens using a method that is relatively expedient and clinically feasible would be desirable. To this end, we developed a clinically-approved approach to isolate endogenous antigen-specific T cells from the peripheral blood for adoptive therapy (“endogenous T cell” or ETC therapy). An unbiased source of T cells would be readily available from the peripheral blood in contrast to TIL therapy (where a surgically accessible tumor with an adequate population of infiltrating lymphocytes is required), In contrast to engineered TCR or CAR, no genetic modification is required and there is greater flexibility in targeting tumor-associated antigens. With ETC therapy, more rapid implementation of clinical studies is also possible since the only requirement is a new GMP-grade peptide whereas specific toxicities and binding properties associated with each new TCR or CAR construct need to be addressed. To demonstrate the feasibility of the form of ETC therapy presented in this study, we target a prototypic cancer testis antigen (CT antigen), NY-ESO-1, expressed in several solid tumor types including breast, colorectal, ovarian, lung cancer and sarcoma.

Patients with soft tissue sarcomas can express very high levels of NY-ESO-1 but the often undetectable levels of NY-ESO-1 specific CD8^+^ T cells in their peripheral blood has made it difficult to generate autologous NY-ESO-1 T cell products for adoptive immunotherapy. Here we demonstrated that after stimulating CD25 depleted T cells from sarcoma patients with autologous dendritic cells pulsed with the NY-ESO-1_157-165_ peptide and *in vitro* exposure to IL-21, rare wells containing measurable tetramer positive T cells can be identified and clinical grade cell sorting can be used to enrich and expand these populations to large numbers of highly purified tumor-reactive T cells. The cell products are oligoclonal, have an effector memory phenotype, and are of sufficient functional avidity to lyse T2 cells pulsed with low concentrations of NY-ESO-1 peptide, and tumor cell lines that express NY-ESO-1.

We were able to obtain regulatory approval from the FDA to use this methodology for the generation of clinical grade T cell products for the treatment of patients with advanced cancer. The selection reagents for cell sorting, peptide-MHC tetramer complexes conjugated to a fluorescence molecule, were tested for sterility, endotoxin and mycoplasma prior to GMP use in order to assure safety in these reagents in a GMP environment. While MHC streptamers (produced by IBA) offer the advantage of reversible binding to the target cell [24,25], there was no apparent impact on the ability to expand these cells after sorting and given the small amount of tetramer reagent used and the multiple washes occurring during the two expansions, any remaining tetramer present in the final product is unlikely to be clinically relevant and was not a concern for regulatory approval.

Other investigators have used retroviral and lentiviral vectors to insert an NY-ESO-1 specific TCR into T cells and redirect their specificity to tumor cells [5,26]. Genetic modification has the advantage of using an off the shelf reagent to engineer each patient’s T cells with an identical high affinity TCR. However, there are theoretical safety concerns related to the potential for cross pairing of the introduced TCR chains with endogenous chains to confer autologous reactivity [11] and the use of affinity-enhanced and xenogeneic TCR has also raised serious safety concerns related to recent patient deaths [8,27]. Practical considerations of genetically modifying cells for altered specificity such as the cost and regulatory burden are also significant issues for many labs. The ETC (endogenous T cell therapy) approach presented here, obviates many of these issues and can provide T cell products that have comparable avidity and function to those generated by transfer of genes that encode a high affinity NY-ESO-1 specific TCR.

For this study, IL-21 was used to enrich for a population of CD8+ T cells with a central memory phenotype [19] which in our previous studies has demonstrated long-term in vivo persistence [28]. Our decision to set a target cell dose of 10^10^ cells/m^2^ was based on prior studies where this dose range has been effective and allows for in vivo tracking of transferred T cells [2,13,29]. In light of the high replicative potential of IL-21 –primed CTL, a lower optimal cell dose may yet be determined; a lower dose requirement would allow for the collection of lower blood volumes and increase the feasibility of generating T cells to multiple targets from a single collection.

It is interesting to speculate on why the two final products tested by deep sequence were both over 85% comprised of a dominant clone and all products consisted of 15 or fewer clones. It may be that T cells specific for NY-ESO-1 are infrequently found in the natural repertoire. It is also likely that certain clones become dominant during the expansions following selection by cell sorting.

This was a small series limited to HLA A*0201 positive patients with NY-ESO-1 expressing sarcomas, accompanied by some variability among the NY-ESO-1 specific T cell products with regards to their lytic ability and expression of markers of T cell differentiation. However, clinical cell sorting with HLA tetramers offers a platform that can be easily adapted to additional MHC restricted targets (for example other CT antigens such as PRAME and MAGE family antigens), and additional HLA alleles. Moreover this approach may be used with other reagents for detecting tumor-reactive T cells such as CD137, where knowledge of the HLA restricting allele and tumor antigen is not necessary [30]. Antigen-specific CD4 T cells have also been shown to be effective for the treatment of patients with solid tumor malignancies and, with further development of the Class II tetramers, it will be possible to rapidly isolate such T cells in future trials [3,31,32].

In summary, the results of this study demonstrate that rare tumor-reactive antigen-specific T cells can be successfully isolated from the peripheral blood of patients and expanded to numbers sufficient for adoptive therapy. By the use of *in vitro* stimulation as a means to augment the population of antigen-specific CTL in culture and enrich for a pool of CD28^hi^ helper-independent CD8+ T cells following exposure to IL-21, together with tetramer-guided cell sorting under clinical manufacturing conditions, it is feasible to generate tumor-reactive antigen-specific CTL of sufficient magnitude (>30 billion) for therapy in 5-6 weeks from PBMC collection to T cell product. Strategies to further streamline this approach and validate the routine generation of T cells against additional target antigens such as WT-1, and additional CT family antigens that are expressed broadly in solid tumors, will facilitate entry of adoptive therapy into mainstream clinical practice and consideration as a possible treatment modality in patients with solid tumor malignancies.

## Conclusion

NY-ESO-1 specific T cells can be isolated and expanded from the peripheral blood of patients with NY-ESO-1 expressing cancers under clinical manufacturing conditions for use in adoptive T cell therapy trials.

## Methods

### Patients, sample acquisition and initial processing

Patients were enrolled on protocols approved by the Fred Hutchinson Cancer Research Center (FHCRC) IRB for tissue and blood procurement between September 2011 and May 2012, and provided informed consent in accordance with the Helsinki Declaration. HLA typing of patient samples was performed at the Puget Sound Blood Center (PSBC). Patients that expressed HLA A*0201, had an ECOG performance status of 1 and tumor biopsies with positive staining for NY-ESO-1 in >25% of cells by immunohistochemistry underwent leukapheresis at either the PSBC or University of Washington General Clinical Research Center. All further processing of the leukapheresis product was performed at FHCRC in the Cell Professing Facility (CPF) under current Good Manufacturing Practice (cGMP) guidelines. A portion of the leukapheresis product (2 × 10^9^ cells) was CD25 depleted on a CliniMACS system using clinical grade CD25 MicroBeads [Miltenyi Biotec, Auburn, CA, USA] to remove CD4^+^ regulatory cells. Both the CD25-depleted mononuclear cell fraction, as well as the remaining unprocessed peripheral blood mononuclear cells (PBMC), were cryopreserved; the latter being used for dendritic cell (DC) generation.

### Culture of dendritic cells

The protocols for DC generation have been described previously [33]. Briefly, PBMC were suspended in AIM-V medium at 3 × 10^6 cells/mL, and then placed in sterile tissue culture dishes at 3 mL/well to separate plastic adherent and non-adherent populations by culture for 1 hour at 37°C. Adherent cells were treated with GM-CSF (800 U/mL) [MP Biomedicals, Santa Ana, CA, USA] and IL-4 (500 U/mL) [R&D Systems, Minneapolis, MN, USA] for 6 days to promote DC differentiation, and the DC were then matured for 2 days using a cytokine cocktail containing TNF-α (10 ng/mL), IL-1β (2 ng/mL), IL-6 (1000 U/mL), PGE-2 (1000 ng/mL) [all from R&D Systems, Minneapolis, MN, USA], IL-4 (500 U/mL) and GM-CSF (800 U/mL). Mature DC’s were generally used fresh (without freezing or thawing) for the initial stimulation but excess DC’s were frozen and frequently used in the second stimulation.

### Stimulation of NY-ESO-1 specific T cells

DC’s were pulsed for 2 hours with 10-40 μg/mL of the NY-ESO-1_157-165_ peptide in PBS with 1% HSA, washed, irradiated (5000 rads), and then co-cultured in 48 well plates with CD25 depleted PBMC in RPMI, 25 mM HEPES, 2 mM L-glutamine, 10% human AB serum (CTL medium) and β2-microglobulin (3 μg/mL) [Scripps Laboratories, San Diego, CA, USA]. Each plate was seeded with 70 × 10^6^ CD25 depleted PBMC and 1.8 to 5 × 10^6^ DC's divided equally among the 48 wells. Two stimulations with peptide pulsed DC were performed 7 days apart. During the first stimulation, IL-21 (30 ng/mL) [Peprotech, Rocky Hill, NJ, USA] was added twice in the first 4 days, and during the second stimulation cycle, IL-21 (30 ng/mL) is added on the first day and IL-2 (10 ng/mL) [Bayer, Terrytown, NY, USA] and IL-7 (5 ng/mL) [R&D Systems, Minneapolis, MN, USA] were added on the first day and days two through four [18]. Stimulations for gp100 specific and MART1-specific T cells were performed using the same protocol using gp100_154-162_ or MART1_27-35_ peptides, respectively.

### Sorting and expansion

After two stimulations, an aliquot of each well was stained with an HLA A*0201 MHC tetramer folded with the NY-ESO-1_157-165_ peptide produced in the Immunologic Monitoring Core Facility at FHCRC. Wells that contained >0.5% tetramer positive cells were pooled, sorted using only forward, side scatter and single color tetramer parameters on a clinically qualified INFLUX cell sorter [Becton Dickenson, Franklin Lakes, NJ, USA] located within the CPF, and expanded using a rapid expansion protocol (REP) in a sterile 25 mL flask using an IL-2 concentration of 50U/mL as previously described [34]. In some cases, wells with <0.5% tetramer positive cells were sorted and expanded if there was clearly a distinct tetramer positive population. After expansion, a sample of the T cells were again stained with anti-CD8 mAb and NY-ESO-1_157-165_ tetramer to determine the degree of enrichment of tetramer positive (tet^+^) cells, and tested in a chromium release assay for lysis of T2 lymphocytes pulsed with NY-ESO-1_157-165_ peptide. A final large volume expansion was performed using either bags [Lifecell, Branchburg, NJ, USA] or GREX flasks [Wilson-Wolf, St. Paul, MN, USA].

To facilitate detailed functional analysis, an aliquot of some T cell products was removed from the CPF after the first expansion, and expanded in the research lab for *in vitro* assays to assess recognition of target cells pulsed with various concentrations of peptide and to assess cytokine production in response to antigen stimulation. Cell surface phenotype and TCR Vβ spectratyping were performed on aliquots from the second expansion.

### Phenotyping and functional analysis of NY-ESO-1-specific T cells

Cell surface phenotype was determined by co-staining with NY-ESO-1 tetramer (APC) and the following fluorochrome conjugated antibodies: CD8 (BV421) [BD Bioscience, Franklin Lakes, NJ, USA], CD28 (PE- Texas Red) [Beckman Coulter, Brea Ca, USA], CD27 (BV711), CCR7 (PE-Cy7), CD127 (PE) [all BD Pharmigen, San Diego, CA, USA], CD62L (APC-eFluor780) [Biolegend, San Diego, CA, USA], and CD45RO (FITC) [Invitrogen, Grand Island, NY]. Cells were analyzed on an LSRII [Becton Dickinson, Franklin Lakes, NJ, USA] using FlowJo software.

The NY-ESO-1 specificity of the expanded T cell products was confirmed using chromium release assay performed on day 12 of the REP. T2 lymphocytes cultured alone for 2 hours or in media with the NY-ESO-1_157-165_ peptide and HLA A*0201 positive, NY-ESO-1 positive tumor line Mel A375 (A375) were used as target cells.

In order to test NY-ESO-1 specific CTL lines in sarcoma tumor lines, the SS cell line SYO-1 (gift of Dr. Akira Kawai [35]) and the MRCL cell line 402 (gift of Pierre Aman [36] ) were used. Because neither of these cell lines are positive for HLA A*0201, they were transfected with the lentivirus pRRLSIN with A*0201 cloned into it. To further purify the A*0201 expressing population, tumor cells were stained using an antibody for A02 (PE) [BD Pharmigen, San Diego, CA, USA] then sorted using a FACSAria cell sorter (Becton Dickson) to isolate A02 expressing tumor cells. Cell were then grown in culture and confirmed to express A02 by flow cytometry prior to chromium release assay.

Target cells were labeled with 100 μCi ^51^Cr in 1 mL of media and co-cultured with effector T cells for 4-6 hours at 37°C plus 5% CO_2_ at a 20:1 effector to target (E:T) ratio unless otherwise specified. Chromium release experiments were performed in sextuplicate and to remove outliers in a consistent fashion, the highest and lowest values for each target/effector ratio were discarded. As a positive control, we tested an NY-ESO-1-specific T cell clone generated in our lab [23], and T cells transduced with the αLY NY-ESO-1 specific TCR [26] (gift of Paul Robbins courtesy of Dr. E.H. Warren).

For cytokine analysis, T cells were incubated overnight at a 20:1 E:T ratio (200,000 effectors with 10,000 targets) in 200 μL CTL media with T2 lymphocytes alone or with T2 lymphocytes pulsed with NY-ESO-1_157-165_ peptide (10 μg/mL) for 2 hours and then washed before plating in the assay. Supernatant was analyzed for using a luminex assay for IFN-gamma (Fisher) and TNF alpha [R&D Systems, Minneapolis, MN, USA].

### Immunohistochemistry

Immunohistochemical staining for all SS and MRCL patients was performed at the University of Washington Pathology lab with mAb specific for NY-ESO-1 (1:100, E978 clone, Life Technologies, Grand Island, NY) using an automated immunostainer (Bond III). Testis was used as a positive control.

### Spectratyping and deep sequencing of T cell receptors

TCR β-chain spectratype analysis was performed by the Immune Monitoring Laboratory at Fred Hutchinson Cancer Research Center according to the protocol developed by Akatsuka et al. [37] Briefly, cDNA was generated from 1-5 × 10^6^ cells and five multiplex PCR reactions were run to amplify the CDR3 variable regions of each of the 23 TCR β-chain families. The PCR products were analyzed by capillary electrophoresis on the 3730 xl DNA analyzer and GeneMapper software [Life Technologies/Applied Biosystems, Van Allen Way, Ca, USA]. TCR Vβ sequencing and normalization was performed by Adaptive Biotechnologies [Seattle, WA, USA].

## Additional file

Additional file 1: Figure S1. Results after 2 stimulations of PBMC from a melanoma patient using DC’s pulsed with the MART-1_27-35_ peptide. **Figure S2.** CD8 and NY-ESO-1 Tetramer staining of fully expanded, clinical grade products. **Figure S3.** Expression of markers of memory phenotype CD45RO, CD27, CD28, CD62L, CCR7, and CD127 (blue) and controls (red). **Figure S4.** IFN gamma and TNF alpha of fully expanded clinical grade products following co-culture with peptide pulsed and un-pulsed T2 lymphocytes. **Figure S5.** Chromium release assay demonstrating specific lysis of the SS tumor line SYO-1 and the MRCL tumor line 402 at a 20:1 effector to target ratio. Because these tumor lines do not express the HLA A*0201, they were transfected with a lentivirus encoding for A*0201, purified for the A02 expressing cells by flow sorting and grown in culture prior to this assay. **Figure S6.** Vβ spectratyping of each fully expanded clinical products. **Figure S7.** T cell products are oligoclonal.

## Abbreviations

A/I: Adiamycin and Ifosfamide
DC: Dendritic Cell
DTIC: Decarbazine
CAR: Chimeric Antigen Receptor
cGMP: current Good Manufacturing Practice
CPF: Cell Processing Facility
CT Antigens: Cancer-Testis Antigens
CTL: Cytotoxic T Lymphocytes
Doxil: Liposomal Doxorubicin
E:T: Effector to Target Ratio
Gem/tax: gemcitabine and docetaxel
GM-CSF: Ganulocyte Macrophage Colony Stimulating Factor
HLA: Human Leukocyte Antigen
IFN: Interferon
PBMC: Peripheral Blood Mononuclear Cell
mAb: Monoclonal antibody
MHC: Major Histocompatibility Complex
MRCL: Myxoid/ Round Cell Liposarcoma
REP: Rapid Expansion Protocol
RTX: Radiation Therapy
SS: Synovial Sarcoma
TCR: T cell Receptor
Tet: MHC tetramer
TNF: Tumor Necrosis Factor
