# Supplementary material for: Tetramer guided, cell sorter assisted production of clinical grade autologous NY-ESO-1 specific CD8+ T cells
Source: J Immunother Cancer. 2014 Oct 14;2:36. doi: 10.1186/s40425-014-0036-y (PMC4196009; doi:10.1186/s40425-014-0036-y)
Supplement: Additional file 1: Figure S1. — Results after 2 stimulations of PBMC from a melanoma patient using DC’s pulsed with the MART-127-35 peptide. Figure S2. CD8 and NY-ESO-1 Tetramer staining of fully expanded, clinical grade products. Figure S3. Expression of markers of memory phenotype CD45RO, CD27, CD28, CD62L, CCR7, and CD127 (blue) and controls (red). Figure S4. IFN gamma and TNF alpha of fully expanded clinical grade products following co-culture with peptide pulsed and un-pulsed T2 lymphocytes. Figure S5. Chromium release assay demonstrating specific lysis of the SS tumor line SYO-1 and the MRCL tumor line 402 at a 20:1 effector to target ratio. Because these tumor lines do not express the HLA A*0201, they were transfected with a lentivirus encoding for A*0201, purified for the A02 expressing cells by flow sorting and grown in culture prior to this assay. Figure S6. Vβ spectratyping of each fully expanded clinical products. Figure S7. T cell products are oligoclonal. [file 40425_2014_36_MOESM1_ESM.pdf]

Supplemental Figure 1

A

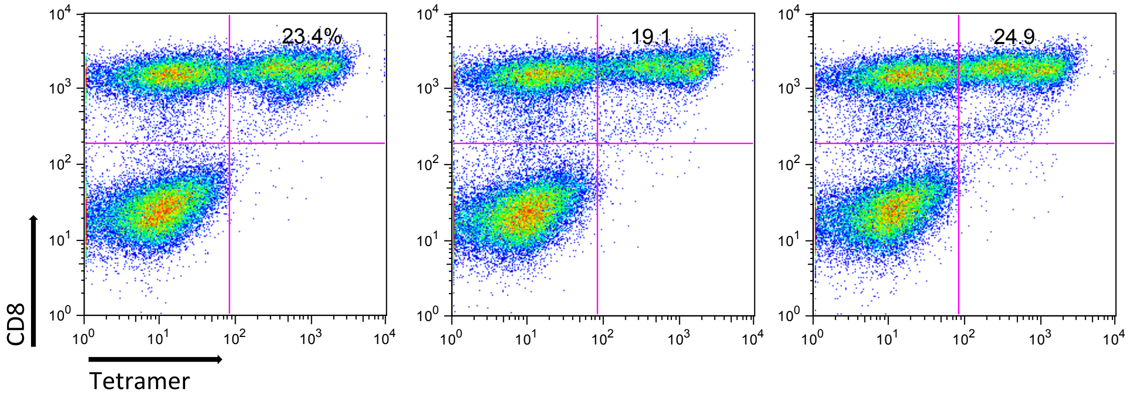

B

	1	2	3	4	5	6
A	2.76	3.8	2.57	3.93	2.53	3.27
B	1.48	3.36	2.59	2.96	3.97	1.94
C	2.22	2.64	2.4	2.38	2.3	4.18
D	2.04	2.38	2.76	4.22	2.37	2.71
E	2.1	3.38	2.87	2.51	5.19	3.32
F	2.47	2.56	3.24	3.09	2.82	2.66
G	2.89	2.3	2.04	2.98	2.23	4.06
H	2.55	3.17	3.58	2.22	2.04	2.41

Supplemental Figure 1: Results after 2 stimulations of PBMC from a melanoma patient using DC's pulsed with the MART-1$_{27\text{-}35}$ peptide.

A) CD8 and tetramer staining of three wells from a 48 well plate are shown. It is not uncommon to have wells with 20% or higher CD8+, tet+ MART-1 specific cells.

B) Percentage CD8+, tetramer+ cells for each well of a 48 well plate are shown following 2 stimulations using MART-1 peptide (M27). Even when high proportions of CD8+, tet+ cells are not seen, there are usually detectable CD8+, tet+ populations in each well of a 48 well plate.

Supplemental Figure 2

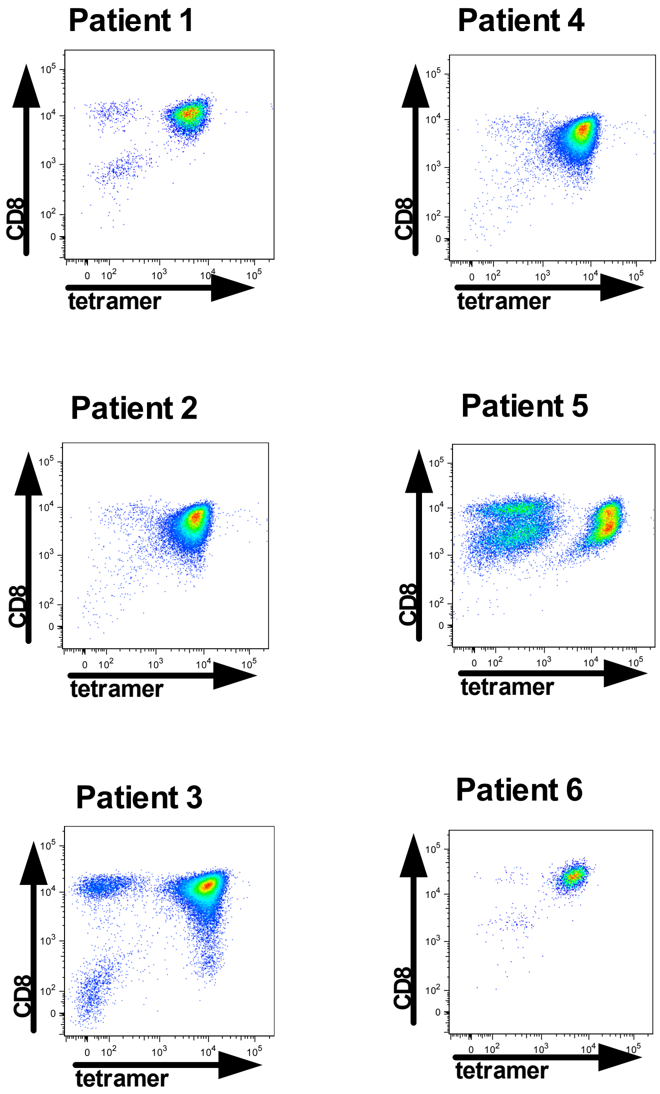

Supplemental Figure 2: CD8 and NY-ESO-1 Tetramer staining of fully expanded, clinical grade products.

Supplemental Figure 3

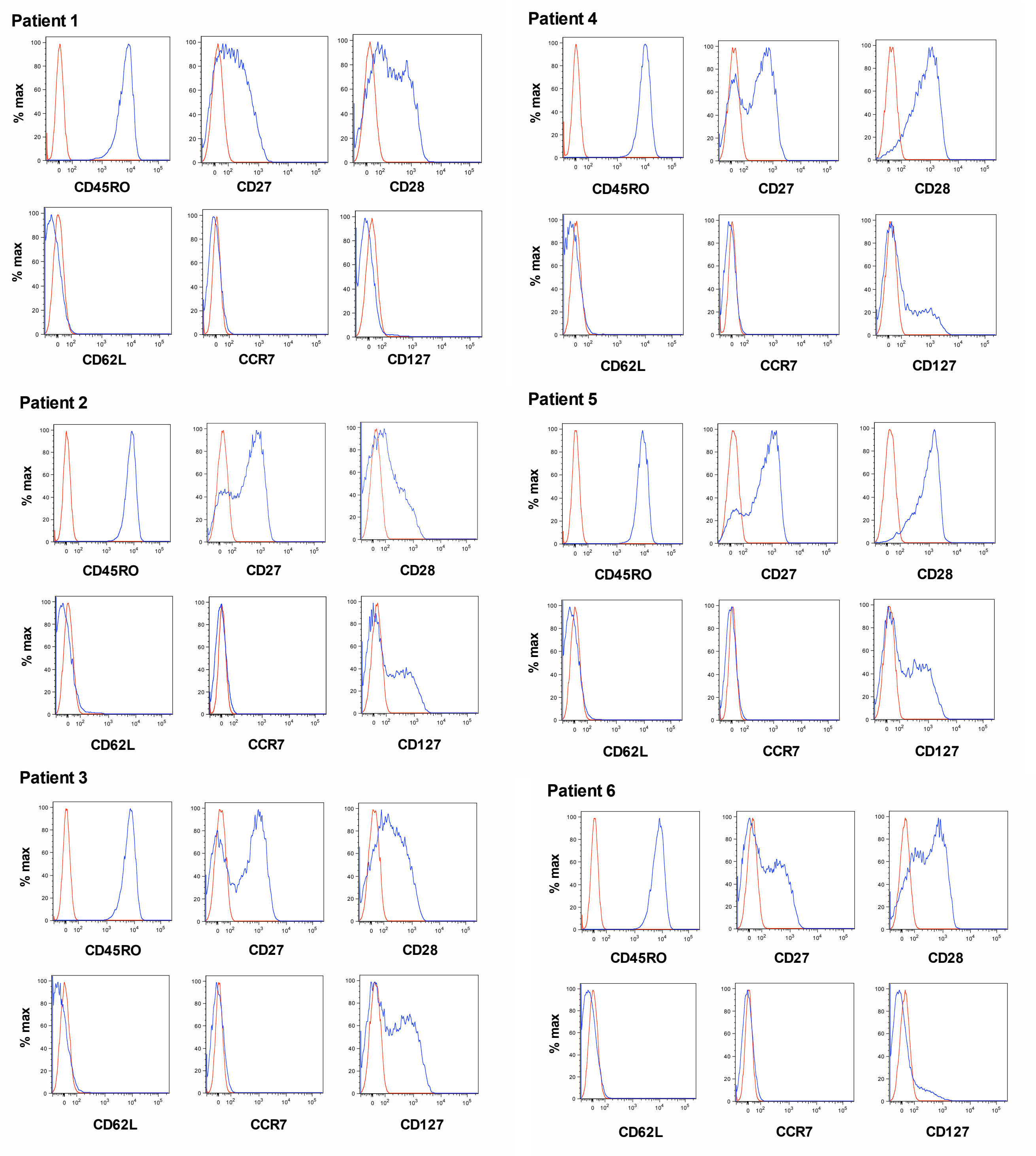

Supplemental Figure 3: Expression of markers of memory phenotype CD45RO, CD27, CD28, CD62L, CCR7, and CD127 (blue) and controls (red).

Supplemental Figure 4

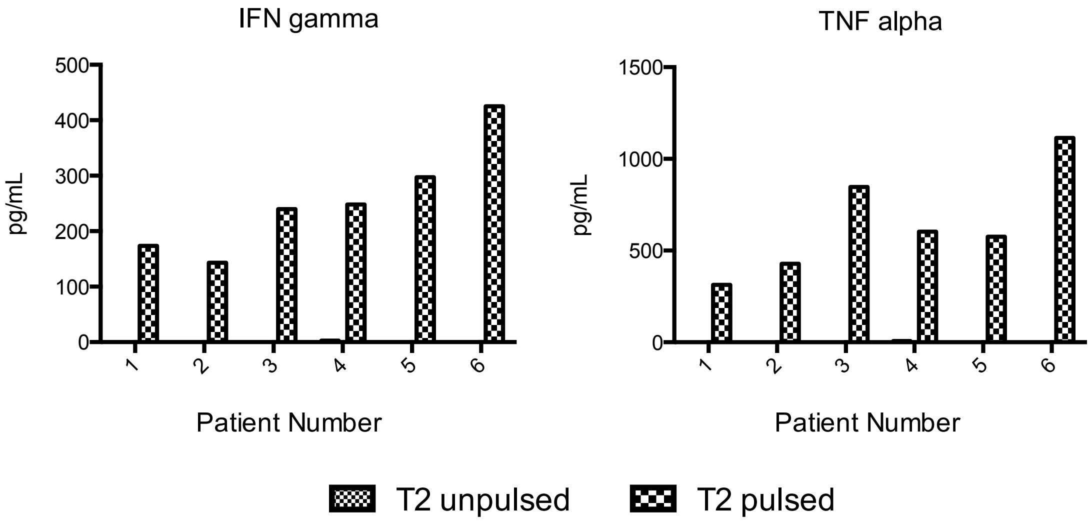

Supplemental Figure 4: IFN gamma and TNF alpha of fully expanded clinical grade products following co-culture with peptide pulsed and un-pulsed T2 lymphocytes.

Supplemental Figure 5

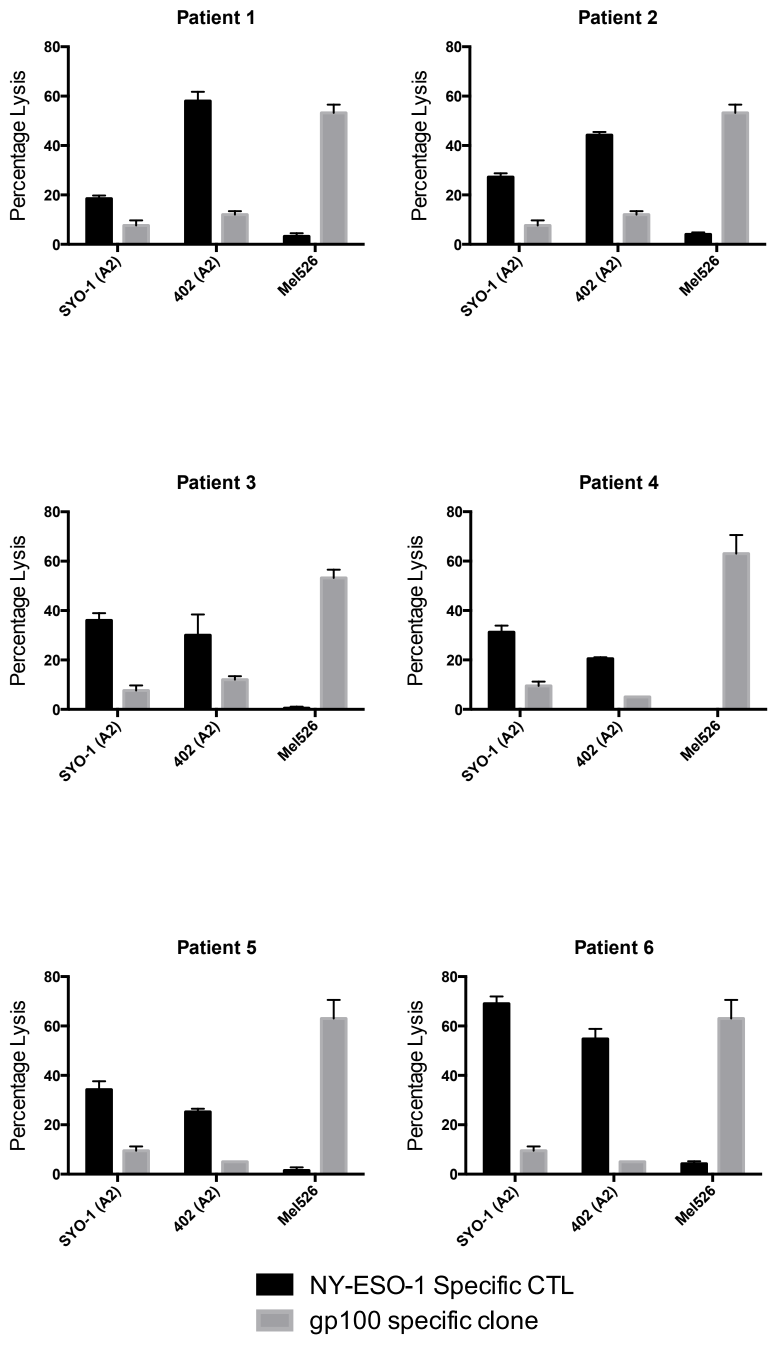

Supplemental Figure 5: Chromium release assay demonstrating specific lysis of the SS tumor line SYO-1 and the MRCL tumor line 402 at a 20:1 effector to target ratio. Because these tumor lines do not express the HLA A*0201, they were transfected with a lentivirus encoding for A*0201, purified for the A02 expressing cells by flow sorting and grown in culture prior to this assay.

Supplemental Figure 6:

Patient 1

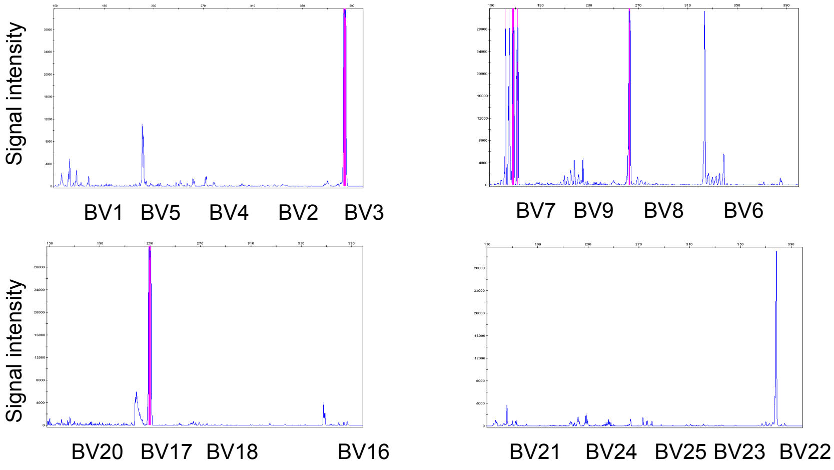

Patient 2

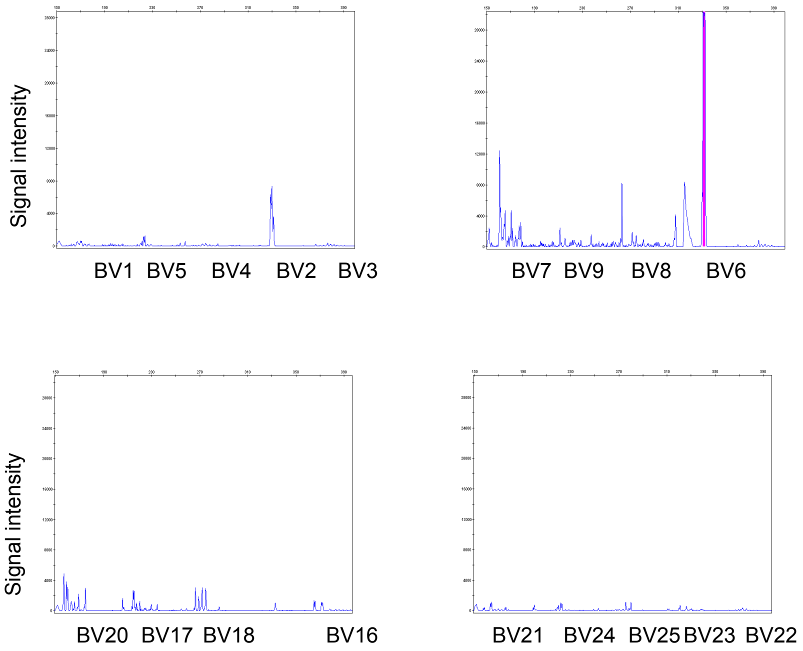

Supplemental Figure 6: Vβ spectratyping of each fully expanded clinical products.

Supplemental Figure 6 (continued)

Patient 3

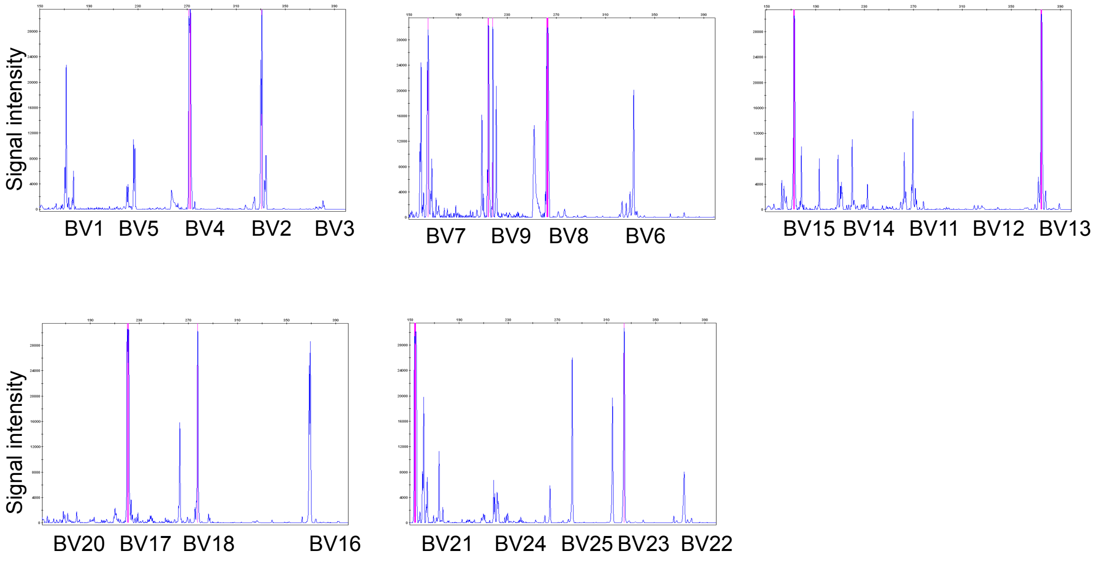

Patient 4

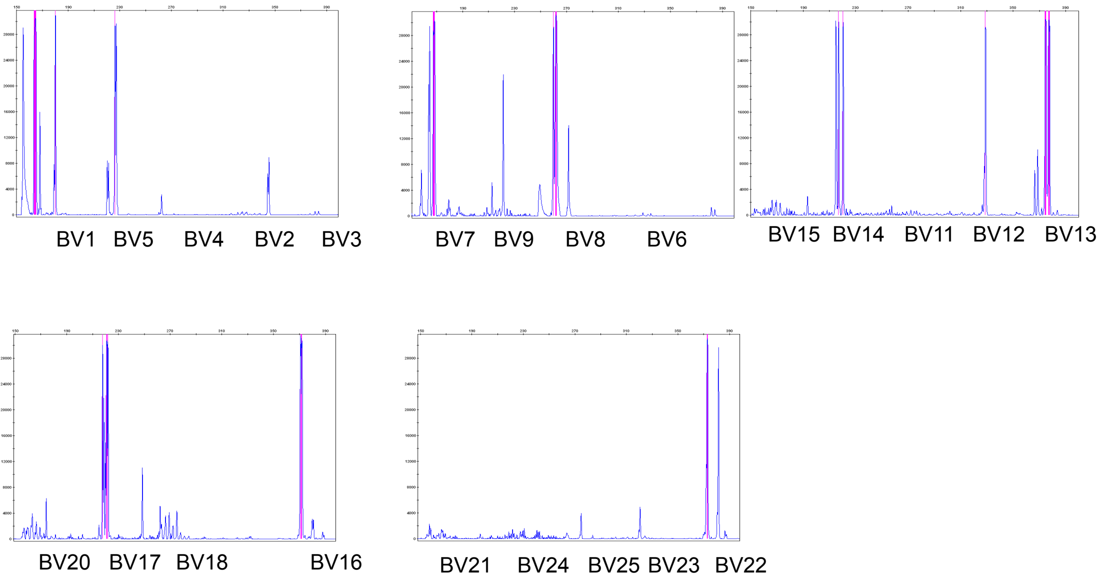

Supplemental Figure 6 (continued)

Patient 5

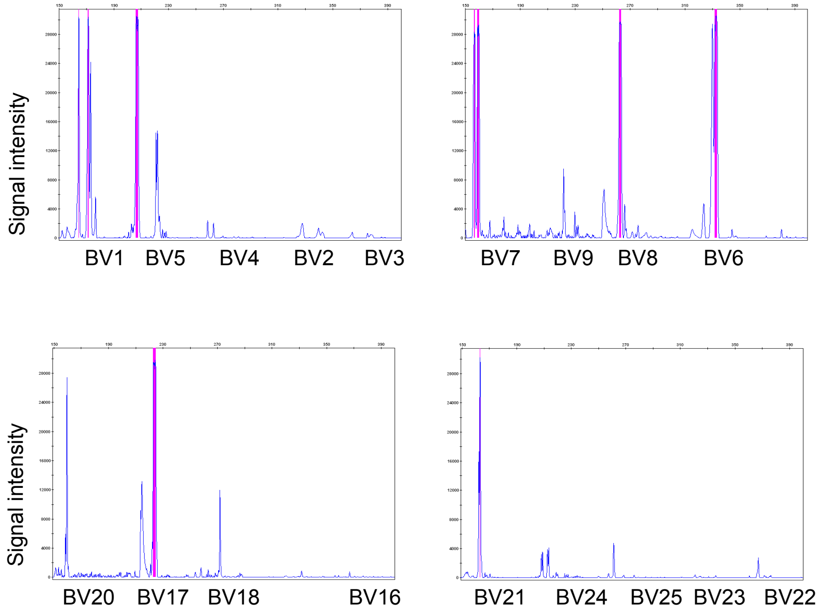

Patient 6

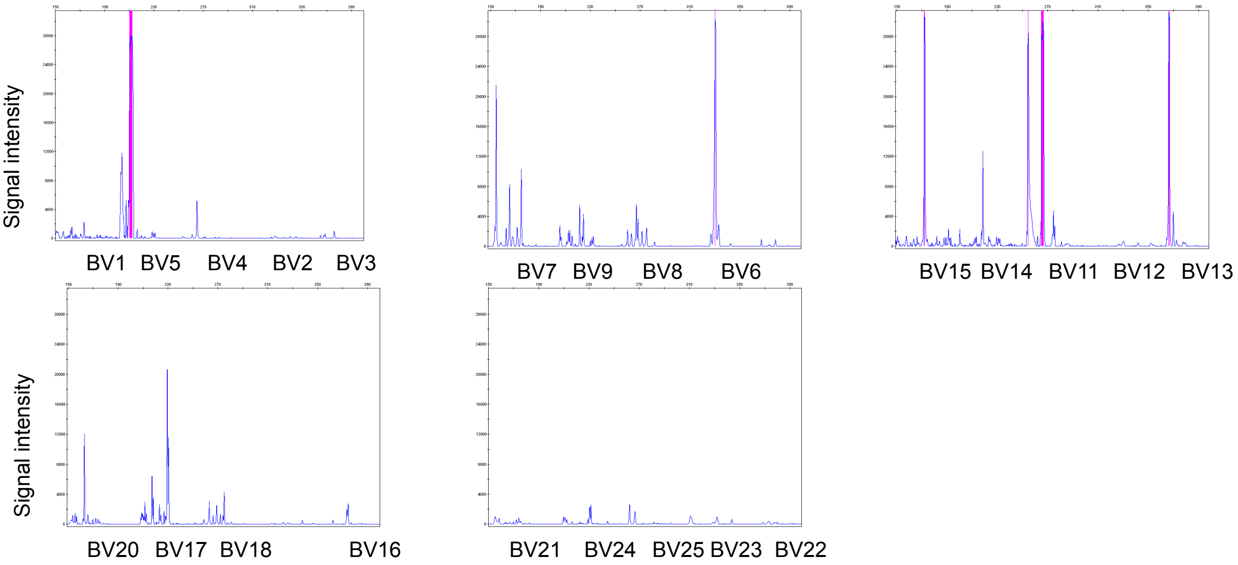

Supplemental Figure 7

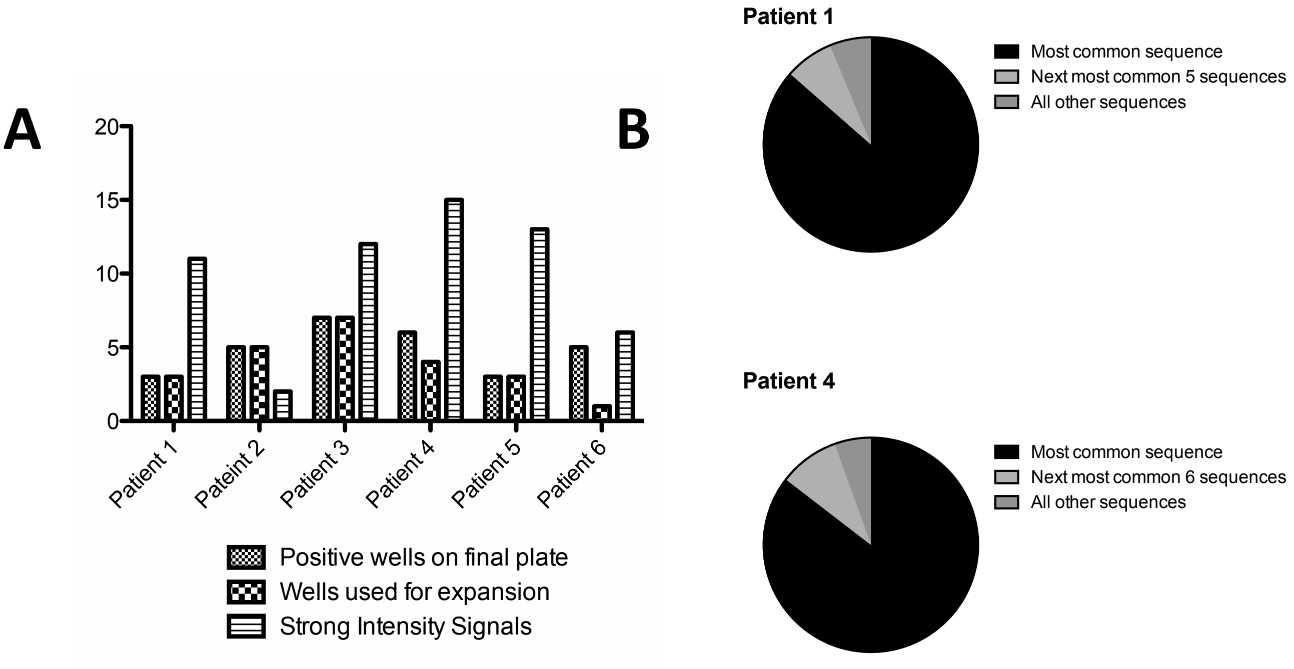

Supplemental Figure 7: T cell products are oligoclonal.

A) Number of dominant peaks on spectratyping of the final product related to the number of positive wells containing NY-ESO-1 specific T cells after DC stimulation and the number of wells used for cell sorting and expansion.

B) Pie chart showing most common sequences seen in the final T cell products for Patients 1 and 4.
